# Association between frailty recovery and dietary variety among community-dwelling older Japanese adults: a longitudinal study from 2023 to 2024

**DOI:** 10.1016/j.jnha.2026.100783

**Published:** 2026-01-21

**Authors:** Tamaki Hirose, Yohei Sawaya, Masahiro Ishizaka, Naori Hashimoto, Akira Kubo, Tomohiko Urano

**Affiliations:** a Department of Physical Therapy, School of Health Sciences, International University of Health and Welfare, Otawara, Tochigi, Japan; b Senior Services Division of Otawara, Otawara, Tochigi, Japan; c Department of Physical Therapy, School of Health Sciences at Odawara, International University of Health and Welfare, Odawara, Kanagawa, Japan; d Department of Geriatric Medicine, School of Medicine, International University of Health and Welfare, Narita, Chiba, Japan

**Keywords:** Frailty, Japan, Dietary variety score, Soybean products, Seaweed

## Abstract

**Background/objectives:**

Although the associations between frailty and nutrition have been reported, no studies have focused on frailty recovery in relation to dietary diversity. This study aimed to clarify the association between frailty recovery and dietary variety, assessed using the Dietary Variety Score (DVS), over a 1-year period.

**Methods:**

A prospective cohort study conducted in City A, Tochigi prefecture, Japan, included 353 individuals aged 73 and 78 years in 2023 who responded to surveys in both 2023 and 2024. The Kihon Checklist (KCL) and DVS were used for assessments. According to the KCL results, participants who were robust in both years or who improved from frailty or pre-frailty in 2023 to robust in 2024 were classified as the “robust-maintained/-recovered group,” and all other participants were classified as “others.” Statistical analyses included group comparisons and binomial logistic regression analysis to examine factors associated with the robust-maintained/-recovered group using DVS categories (high/low) or ten individual food groups as independent variables.

**Results:**

The robust-maintained/-recovered group had a higher frequency of egg, soybean, soybean product, seaweed, potato, and fruit consumption. Furthermore, High DVS at baseline was significantly associated with the robust-maintained/-recovered group (odds ratio = 2.32, p < 0.001). A similar association was observed for soybean products (odds ratio = 1.83, p = 0.008).

**Conclusions:**

These findings suggest that sustained dietary diversity may be an effective strategy for recovery from frailty in older adults. Specifically, intake of traditional Japanese foods such as soybean products and “small food add-ons” such as seaweeds and fruits may offer a valuable approach to its management.

## Highlights

• Frailty recovery examined in relation to dietary diversity over 1 year.
• High DVS was consistently associated with robust or recovered frailty status.
• Sustained dietary diversity may aid frailty management in older adults.
• “Small food add-ons” of soy, seaweed, and fruit may help prevent frailty.

## 1 Introduction

Extension of healthy life expectancy and narrowing of the gap between life expectancy and healthy life expectancy are critical goals for achieving a healthy aging society worldwide. One key factor involved in achievement of these goals is “frailty” [1], which is considered an intermediate stage between robustness and disability and is characterized by the reversible involvement of physical, psychological, and social domains [[2], [3], [4]]. Prevention and early intervention are important for both individuals and society.

Nutrition is a key factor for addressing frailty. In large-scale surveys targeting community-dwelling older adults or routine intervention settings, there is a need for simple and practical tools to assess their nutritional status. One such tool is the Dietary Variety Score (DVS) [5], which assesses dietary diversity based on the habitual intake frequency of ten food groups that constitute the main and side dishes. It can be assessed using a questionnaire without the need for special equipment or laboratory tests, making it a simple and accessible method for evaluating dietary variety [[5], [6], [7]].

Numerous studies targeting community-dwelling older adults in Japan have reported associations between DVS and adverse health outcomes. Cross-sectional studies have shown that low dietary diversity is associated with frailty [8,9], oral frailty [10], and sarcopenia [11]. These trends have also been observed during the COVID-19 pandemic [12,13]. Similar associations between low dietary diversity and adverse health outcomes have been reported worldwide [[14], [15], [16]]. In longitudinal studies, the combination of high physical activity levels and dietary diversity has been associated with a significantly lower incidence of new-onset frailty [17]. Furthermore, dietary variety has been linked to frailty components such as the maintenance of handgrip strength and gait speed, as well as a reduced risk of dementia [6,18]. However, to date, no studies have examined frailty “recovery” as an outcome—a concept regarded as reversible—using DVS as a simple assessment tool.

In this study, we aimed to clarify the association between dietary diversity, assessed using the DVS, and recovery from frailty over a 1-year period from 2023 to 2024, which was set as the primary outcome.

## 2 Methods

### 2.1 Study design and setting

This was a 1-year longitudinal study conducted between 2023 and 2024. A written explanation of the questionnaire was provided to all participants, who were notified that completing it would be considered as providing informed consent. The Ethics Committee of the International University of Health and Welfare approved this study (approval numbers 22-Io-25 and 21-Io-38-2), which was conducted in accordance with the principles of the Declaration of Helsinki.

This study was conducted as a follow-up to our previous studies [19,20] and involved older individuals residing in City A, Tochigi Prefecture, who were recruited using a mail-in questionnaire survey. The target municipality has a population density distribution similar to the national distribution in Japan, with an aging rate of approximately 30%; thus, the demographic characteristics are comparable with those of community-dwelling older adults in Japan [19,20]. In 2023, we targeted individuals aged 73 and 78 years among the 519 individuals who responded in 2023. Consequently, 353 individuals with available data for 2023 and 2024 were included in the analysis (Figure S1).

### 2.2 Frailty assessment

Frailty was assessed using the Kihon Checklist (KCL). As shown in earlier research, a total score of 0–3 points was classified as robust, 4–7 points as pre-frailty, and 8 points or more as frailty [21,22].

The participants were classified into two groups based on frailty status transitions, as assessed in 2023 and 2024. This classification is based on the findings of a previous study [20].

### 2.3 Longitudinal grouping based on changes in frailty status

The following two pattern classifications were implemented in accordance with previous studies [23,24].

• Main pattern: Classification based on recovery in frailty status

Participants were classified into robust-maintained/-recovered group (n = 184) and others (n = 169) based on frailty status recovery. The robust-maintained/-recovered group included individuals who were classified as robust in both 2023 and 2024, as well as those who were classified as pre-frail or frail in 2023 but improved to robust in 2024. All other participants were assigned to the ‘others’ group.

• Sub-pattern: Classification based on deterioration in frailty status

Participants were classified into frailty-persistent/new-onset group (n = 64) and others (n = 289) based on deterioration in frailty status. The frailty persistent/new-onset group included individuals who were classified as frail in both 2023 and 2024 as well as those who were classified as robust or pre-frail in 2023 but deteriorated to frailty in 2024. All the other participants were classified into the others group.

### 2.4 DVS

Dietary diversity was assessed using the DVS [5]. The frequency of consumption over the past week was evaluated for the following ten food groups: fish and shellfish, meat, eggs, milk, soybean products, green and yellow vegetables, seaweed, potatoes, fruits, and fats and oils. A score of 1 point was assigned for “Eating almost every day,” and 0 points were assigned for “Eating once every 2 days,” “Eating 1–2 days a week,” or “Hardly ever.” The total score, ranging from 0 to 10, was used as a measure of dietary diversity. Of the 10 food groups, fish and shellfish, meat, eggs, milk, and soybean products were classified as high-protein foods, whereas green and yellow vegetables, seaweed, potatoes, fruits, and fats and oils were classified as low-protein foods [11,25].

DVS in both 2023 and 2024 were classified as either high (≥4 points) or low (≤3 points), following the criteria used in prior studies [[5], [6], [7]]. For the primary analysis in this study, the participants were classified into two groups (Low DVS and High DVS) based on the baseline (2023) DVS. For secondary analyses, the participants were further stratified into four groups according to their DVS in 2023 and 2024.

### 2.5 Statistical analyses

Baseline characteristics were compared using the chi-square or Fisher’s exact test. Additionally, differences in the proportions of participants consuming each of the 10 food groups in the DVS, as well as differences in the total score, were conducted using the chi-square or Fisher’s exact test and the Mann–Whitney U test. We confirmed that the total DVS was not normally distributed.

Binomial logistic regression analysis was conducted. The dependent variables were the robust-maintained/-recovered group (1) and others (0). The independent variables were examined using three approaches. In the first approach, the independent variables were the two DVS groups based on baseline data (reference group: Low DVS). In the second approach, the independent variables were the four DVS groups derived from baseline and follow-up data (reference group: group with Low DVS in both 2023 and 2024). These analysis were performed using the forced-entry method. In the third approach, the independent variables were the ten individual food items of the DVS, with variable selection performed using a stepwise method. All multivariable analyses were performed using two models: a crude model and adjusted Model I. The covariates were age, sex, body mass index, living alone, hypertension, hyperlipidemia, cancer, hobbies, and community activities. The same analyses conducted for the main pattern were performed for the sub-pattern. All analyses were performed using SPSS Statistics version 27 (IBM), and a significance threshold of 5% was applied.

## 3 Results

### 3.1 Robust-maintained/-recovered group and others

Table S1 presents a comparison of the baseline characteristics. Comparisons of the proportion of participants consuming each of the 10 food groups and the total DVS revealed significant differences for eggs, soybean products, seaweed, potatoes, fruits, and DVS (total points), as shown in Table 1.

**Table 1:** Comparison of intake proportions of the 10 food groups and total Dietary Variety Scores between groups.

		Robust-maintained/-recovered group (n = 184)	Others (n = 169)	P value
1	Fish/shellfish			
	Almost every day/Once every 2 days/1–2 days a week/Hardly ever	31.0/34.8/33.2/1.1	21.9/30.8/44.4/3.0	0.055
2	Meat			
	Almost every day/Once every 2 days/1–2 days a week/Hardly ever	31.5/42.4/25.5/0.5	23.1/42.6/32.0/2.4	0.140
3	Eggs			
	Almost every day/Once every 2 days/1–2 days a week/Hardly ever	40.8/37.5/20.1/1.6	30.2/30.8/36.1/3.0	0.004*
4	Milk			
	Almost every day/Once every 2 days/1–2 days a week/Hardly ever	47.8/9.8/21.2/21.2	42.6/13.6/18.9/24.9	0.498
5	Soybean products			
	Almost every day/Once every 2 days/1–2 days a week/Hardly ever	64.1/21.7/14.1/0.0	48.5/24.3/25.4/1.8	0.003*
6	Green and yellow vegetables			
	Almost every day/Once every 2 days/1–2 days a week/Hardly ever	73.4/15.2/10.3/1.1	60.4/23.1/15.4/1.2	0.062
7	Seaweeds			
	Almost every day/Once every 2 days/1–2 days a week/Hardly ever	24.5/30.4/42.4/2.7	16.6/29.6/44.4/9.5	0.023*
8	Potatoes			
	Almost every day/Once every 2 days/1–2 days a week/Hardly ever	14.7/27.7/53.8/3.8	8.3/19.5/59.8/12.4	0.002*
9	Fruits			
	Almost every day/Once every 2 days/1–2 days a week/Hardly ever	35.9/15.8/41.3/7.1	26.0/25.4/32.5/16.0	0.002*
10	Fats/oils			
	Almost every day/Once every 2 days/1–2 days a week/Hardly ever	37.5/26.1/33.7/2.7	28.4/37.9/32.5/1.2	0.069
DVS (total points)		4.0 [2.0–6.0]	3.0 [2.0–4.0]	<0.001*

DVS: Dietary Variety Score.

%. Median [25th–75th percentile]. *: p < 0.05.

Robust-maintained/-recovered group: Individuals who were classified as robust in both 2023 and 2024, as well as those who were classified as pre-frailty or frailty in 2023 and transitioned to robust in 2024.

Others: Individuals other than those in the robust-maintained/-recovered group.

The binomial logistic regressions results demonstrated that High DVS at baseline and High DVS in both 2023 and 2024 were significantly associated with the robust-maintained/-recovered group in both the unadjusted model and model I (Table 2 and Table S2). Analysis of the ten food items of DVS revealed that consumption of soy products almost daily at baseline was significantly associated with the robust-maintained/-recovered group (Table 3).

**Table 2:** Association between the robust-maintained/-recovered group and baseline Dietary Variety Score.

		Crude model			Model Ⅰ		
	n	OR	95%CI (Min–Max)	P value	OR	95%CI (Min–Max)	P value
Low DVS (Baseline)	190	Ref			Ref		
High DVS (Baseline)	163	2.31	1.50–3.55	<0.001*	2.32	1.48–3.65	<0.001*

CI: confidence interval, DVS: Dietary Variety Score, Max: maximum, Min: minimum, OR: odds ratio. *: p < 0.05.

The dependent variables were defined as 0 (others) or 1 (robust-maintained/-recovered).

The independent variables were defined as 0 (Low DVS) or 1 (High DVS).

High DVS: DVS ≥ 4; low DVS: DVS ≤ 3.

Model I (adjusted variables): Age, sex, body mass index, living alone, hypertension, hyperlipidemia, cancer, hobbies, and community activities.

Robust-maintained/-recovered group: Individuals who were classified as robust in both 2023 and 2024, as well as those who were classified as pre-frailty or frailty in 2023 and transitioned to robust in 2024.

Others: Individuals other than those in the robust-maintained/-recovered group.

**Table 3:** Association between the robust-maintained/-recovered group and the ten individual food items of the Dietary Variety Score at baseline.

	Crude model			Model Ⅰ		
	OR	95%CI (Min–Max)	P value	OR	95%CI (Min–Max)	P value
Soybean products (Baseline)	1.90	1.24–2.91	0.003*	1.83	1.17–2.86	0.008*

CI: confidence interval, Max: maximum, Min: minimum, OR: odds ratio. *: p < 0.05.

The dependent variables were defined as 0 (others) or 1 (robust-maintained/-recovered).

The independent variables were the ten food items of the Dietary Variety Score, which were entered into the model using a stepwise method. Each food item was coded as 0 (once every 2 days, 1–2 days per week, or hardly ever) or 1 (almost every day).

Model I (adjusted variables): Age, sex, body mass index, living alone, hypertension, hyperlipidemia, cancer, hobbies, and community activities.

Robust-maintained/-recovered group: Individuals who were classified as robust in both 2023 and 2024, as well as those who were classified as pre-frailty or frailty in 2023 and transitioned to robust in 2024.

Others: Individuals other than those in the robust-maintained/-recovered group.

### 3.2 Sub-analysis: Frailty-persistent/new-onset group and others

The results of the baseline between-group comparisons and binomial logistic regression analyses are presented in Tables S3–S7.

## 4 Discussion

This study is novel as, unlike previous research that has mainly focused on the prevention of frailty onset, it examined the association of the latter with frailty recovery. Furthermore, the findings underscore the importance of foods rooted in Japanese dietary culture, such as soybean products, seaweeds, and fruits, including easily adoptable dietary behaviors, such as “small food add-ons,” in daily life. These insights can be utilized to support behavioral changes in residents. The alignment of these findings with food group-based dietary improvements recommended in Japan’s “Health Japan 21” initiative also underscores their public-health significance. Although frailty is often deemed to worsen in a unidirectional manner, it is a reversible condition [3]. Longitudinal observational studies in community-dwelling older adults have demonstrated dynamic and bidirectional transitions [26,27]. Accordingly, the grouping strategy used in this study, frailty status transitions, and the follow-up period focusing on frailty and nutrition were comparable with those reported in previous studies [12,13,[27], [28], [29], [30]]. International evidence indicating that frailty can be reduced in a proportion of older adults supports the findings of the present study.

In this study, individuals with a better nutritional status were more likely to recover to a robust state, whereas those with a poorer nutritional status tended to deteriorate into frailty. These findings suggest that dietary diversity may contribute not only to the prevention of frailty progression but also to improvement in robustness. Furthermore, among the participants in this study, there were no significant differences in body size (e.g., BMI) or background factors (e.g., living alone staus and comorbidities), supporting the notion that dietary diversity itself has a positive impact on health. DVS is considered an index that can capture not only the variety of foods consumed but also the frequency of intake. A study using the Food Frequency Score reported that low dietary diversity, defined as a score <16 out of 30, was associated with physical frailty [31]. According to Weng et al., individuals with low dietary variety have a 1.6-fold higher risk of frailty than those with high dietary variety [32], indicating the potential effectiveness of promoting dietary diversity in frailty prevention. These international findings support our assertion that dietary “colorfulness” and “balance,” as indicators of diversity, play an important role in preventing frailty.

Contrary to our expectations, a comparison of individual food items revealed that recovery to a robust state and progression to frailty were more closely linked to the consumption of soybean products, seaweed, potatoes, and fruits than to the consumption of protein-rich foods such as meat and eggs. Among these, soybean products such as miso, tofu, and natto are representative of traditional Japanese cuisine. Soybean products contain isoflavones, are rich in plant-based proteins and low in cholesterol. Their contribution to the maintenance of health in older adults has been reported [33,34], potentially supporting the findings of the present study. Research conducted among community-dwelling older adults in Japan found that greater adherence to a dietary pattern emphasizing foods typical of the Japanese diet, such as fish, soybean products, vegetables, and fruits, is associated with a lower prevalence of sarcopenia in both men and women [35]. International research has demonstrated that greater adherence to the Mediterranean diet is associated with a lower risk of frailty [36,37]. These findings suggest that nutritionally balanced traditional dietary habits in specific regions may prevent frailty. With regard to fruit, several reports, including review studies of older adults overseas, have shown that adequate intake or high consumption is associated with a lower risk of frailty onset [38,39]. In contrast, seaweed rarely appears on the table, unless intentionally included, suggesting that dietary monotony may be associated with frailty. In this context, making a conscious effort to incorporate food such as seaweed and fruits as “small food add-ons” in daily meals may be an effective approach to enhancing dietary diversity.

In addressing frailty, not only nutrition but also physical activity and social participation are considered important components [19,20,[40], [41], [42]]. However, the evidence related to nutrition and frailty remains limited. Even in the International Conference on Frailty and Sarcopenia Research guidelines, items concerning nutrition are rated as having low evidence levels and are only conditionally recommended [43]. Furthermore, nutrition has the advantage of being relatively easy for older adults to implement and sustain in daily life.

In addition, impaired autonomic nervous system function has been reported to be associated with declines in instrumental activities of daily living [44], which may indirectly relate to reduced dietary intake through mechanisms such as decreased energy expenditure and appetite. Therefore, the development of more effective and multifaceted approaches that combine nutrition, physical activity, and social participation is required.

This study has several limitations. First, the survey was limited to two specific age groups and was conducted as part of a longitudinal follow-up; this may have restricted the sample size and introduced selection bias, thereby limiting the generalizability of the findings. Second, because the study was conducted within the framework of a municipal survey using a mail-based questionnaire, the scope of the survey items was limited and the follow-up period was restricted to 1 year. In addition, detailed information on social background, hobbies, and prior interventions (such as exercise or nutritional guidance) could not be obtained. In addition, as this was an observational study without any planned interventions such as physical therapy or exercise, caution is warranted when interpreting improvements in frailty status. Third, the Food Frequency Questionnaire that assesses dietary intake amounts was not used, and biochemical data were not obtained. However, nutrition is one of the three main pillars [40] and a key component of frailty prevention. Hence, dietary approaches such as the “small food add-ons” identified in this study may represent a feasible and sustainable nutritional intervention option for older adults, even in the absence of other specialized interventions. In future, intervention studies incorporating quantitative assessments of total protein and energy intakes are warranted.

## 5 Conclusions

These findings indicate that maintaining dietary diversity may contribute not only to prevention but also to recovery from frailty. In particular, conscious dietary behaviors involving traditional Japanese foods such as soybean products, as well as “small food add-ons” such as seaweed and fruits, may serve as a practical strategy to support older adults and combat frailty.

## CRediT authorship contribution statement

Conceptualization: TH, YS, and TU. Data curation: TH, YS, MI, and NH. Formal analysis: YS. Funding acquisition: TH, YS, and TU. Investigation: TH, YS, MI, NH, and AK. Methodology: TH, YS, and TU. Project administration: TH, MI, NH, and AK. Resources: TH, MI, and NH. Software: TH and YS. Supervision: AK and TU. Validation: TH and YS. Visualization: TH and YS. Writing the original draft: TH and YS. TU. Writing, review, and editing: All authors.

## Declaration of Generative AI and AI-assisted technologies in the writing process

The authors used AI-assisted technologies only for spelling and grammatical corrections in limited portions of the manuscript. All content was subsequently reviewed, edited, and approved by the authors to ensure accuracy.

## Funding

This study was funded by the JSPS Grants-in-Aid for Scientific Research (grant numbers 25K14197, 22K17539, and 23K06873) and a JGS Grant for Geriatric Nutrition Research supported by the Otsuka Pharmaceutical Factory.

## Data statement

Research data is not shared because of privacy protection of City Hall provisions.

## Declaration of competing interest

None.

## Glossary

DVS: Dietary variety score
KCL: Kihon checklist
GLIM: Global leadership initiative on malnutrition
BMI: Body mass index

## References

[1] Satake S., Arai H. (2020). Chapter 1 frailty: definition, diagnosis, epidemiology. Geriatr Gerontol Int.

[2] Tanaka T., Takahashi K., Hirano H., Kikutani T., Watanabe Y., Ohara Y. (2018). Oral frailty as a risk factor for physical frailty and mortality in community-dwelling elderly. J Gerontol A Biol Sci Med Sci.

[3] Pollack L.R., Litwack-Harrison S., Cawthon P.M., Ensrud K., Lane N.E., Barrett-Connor E. (2017). Patterns and predictors of frailty transitions in older men: the osteoporotic fractures in men study. J Am Geriatr Soc.

[4] Trevisan C., Veronese N., Maggi S., Baggio G., Toffanello E.D., Zambon S. (2017). Factors influencing transitions between frailty states in elderly adults: the Progetto Veneto Anziani longitudinal study. J Am Geriatr Soc.

[5] Kumagai S., Watanabe S., Shibata H., Amano H., Fujiwara Y., Shinkai S. (2003). Effects of dietary variety on declines in high-level functional capacity in elderly people living in a community. Nihon Koshu Eisei Zasshi.

[6] Yokoyama Y., Nishi M., Murayama H., Amano H., Taniguchi Y., Nofuji Y. (2017). Dietary variety and decline in lean mass and physical performance in community-dwelling older Japanese: a 4-year follow-up study. J Nutr Health Aging.

[7] Momoki C., Habu D., Ogura J., Tada A., Hasei A., Sakurai K. (2017). Relationships between sarcopenia and household status and locomotive syndrome in a community-dwelling elderly women in Japan. Geriatr Gerontol Int.

[8] Hayakawa M., Motokawa K., Mikami Y., Yamamoto K., Shirobe M., Edahiro A. (2021). Low dietary variety and diabetes mellitus are associated with frailty among community-dwelling older Japanese adults: a cross-sectional study. Nutrients.

[9] Motokawa K., Watanabe Y., Edahiro A., Shirobe M., Murakami M., Kera T. (2018). Frailty severity and dietary variety in Japanese older persons: a cross-sectional study. J Nutr Health Aging.

[10] Hoshino D., Hirano H., Edahiro A., Motokawa K., Shirobe M., Watanabe Y. (2021). Association between oral frailty and dietary variety among community-dwelling older persons: a cross-sectional study. J Nutr Health Aging.

[11] Kiuchi Y., Doi T., Tsutsumimoto K., Nakakubo S., Kurita S., Nishimoto K. (2023). Association between dietary diversity and sarcopenia in community-dwelling older adults. Nutrition.

[12] Yokoro M., Otaki N., Yano M., Imamura T., Tanino N., Fukuo K. (2023). Low dietary variety is associated with incident frailty in older adults during the coronavirus disease 2019 pandemic: a prospective cohort study in Japan. Nutrients.

[13] Kinoshita K., Satake S., Arai H. (2022). Impact of frailty on dietary habits among community-dwelling older persons during the COVID-19 pandemic in Japan. J Frailty Aging.

[14] Verlaan S., Ligthart-Melis G.C., Wijers S.L.J., Cederholm T., Maier A.B., de van der Schueren M.A.E. (2017). High prevalence of physical frailty among community-dwelling malnourished older adults-A systematic review and meta-analysis. J Am Med Dir Assoc.

[15] Konglevoll D.M., Andersen L.F., Thoresen M., Totland T.H., Hopstock L.A., Hjartåker A. (2024). Dietary trajectories over 21 years and frailty in Norwegian older adults: the Tromso Study 1994-2016. Eur J Nutr.

[16] Wang X.M., Zhong W.F., Zhang Y.T., Xiang J.X., Chen H., Li Z.H. (2024). Association between dietary diversity changes and frailty among Chinese older adults: findings from a nationwide cohort study. Nutr J.

[17] Osuka Y., Kojima N., Yoshida Y., Kim M., Won C.W., Suzuki T. (2019). Exercise and/or dietary varieties and incidence of frailty in community-dwelling older women: a 2-year cohort study. J Nutr Health Aging.

[18] Yokoyama Y., Nofuji Y., Seino S., Abe T., Murayama H., Narita M. (2023). Association of dietary variety with the risk for dementia: the Yabu cohort study. Public Health Nutr.

[19] Hirose T., Sawaya Y., Ishizaka M., Hashimoto N., Kubo A., Urano T. (2022). Kihon Checklist items associated with the development of frailty and recovery to robust status during the COVID-19 pandemic. Geriatr Gerontol Int.

[20] Hirose T., Sawaya Y., Ishizaka M., Hashimoto N., Kubo A., Urano T. (2024). Prevalence and factors associated with changes in frailty among community-dwelling older adults in Japan during the COVID-19 pandemic: a prospective cohort study from 2020 to 2022. Geriatr Gerontol Int.

[21] Satake S., Senda K., Hong Y.J., Miura H., Endo H., Sakurai T. (2016). Validity of the Kihon checklist for assessing frailty status. Geriatr Gerontol Int.

[22] Satake S., Shimokata H., Senda K., Kondo I., Toba K. (2017). Validity of Total Kihon Checklist Score for predicting the incidence of 3-year dependency and mortality in a community-dwelling older population. J Am Med Dir Assoc.

[23] Yuan Y., Li N.S., Huang X., Li N., Zheng J., Huang F. (2022). The identification and prediction of frailty based on Bayesian network analysis in a community-dwelling.older population. BMC Geriatr.

[24] Zanforlini B.M., Trevisan C., Bertocco A., Piovesan F., Dianin M., Mazzochin M. (2019). Phase angle and metabolic equivalents as predictors of frailty transitions in advanced age. Exp Gerontol.

[25] Budhathoki S., Sawada N., Iwasaki M., Yamaji T., Goto A., Kotemori A. (2019). Association of animal and plant protein intake with all-cause and cause-specific mortality in a Japanese cohort. JAMA Intern Med.

[26] Kojima G., Taniguchi Y., Iliffe S., Jivraj S., Walters K. (2019). Transitions between frailty states among community-dwelling older people: A systematic review and meta-analysis. Ageing Res Rev..

[27] Ohashi M., Yoda T., Imai N., Fujii T., Watanabe K., Tashi H. (2021). Five-year longitudinal study of frailty prevalence and course assessed using the Kihon checklist among community-dwelling older adults in Japan. Sci Rep.

[28] Setiati S., Laksmi P.W., Aryana I.G.P.S., Sunarti S., Widajanti N., Dwipa L. (2019). Frailty state among Indonesian elderly: prevalence, associated factors, and frailty state transition. BMC Geriatr.

[29] Yokoyama H. (2025). Impact of abdominal obesity on frailty development: a web-based survey using a smartphone health app. Geriatrics (Basel).

[30] Kiesswetter E., Pohlhausen S., Uhlig K., Diekmann R., Lesser S., Uter W. (2014). Prognostic differences of the Mini Nutritional Assessment short form and long form in relation to 1-year functional decline and mortality in community-dwelling older adults receiving home care. J Am Geriatr Soc.

[31] Kiuchi Y., Makizako H., Nakai Y., Tomioka K., Taniguchi Y., Kimura M. (2021). The association between dietary variety and physical frailty in community-dwelling older adults. Healthcare (Basel).

[32] Weng S.E., Lai H.Y., Chen I.T., Kuo M.H., Chen L.K., Hsiao F.Y. (2024). Diverse diets, resilient aging: a systematic review and meta-analysis on the protective role of dietary diversity against frailty in older adults. Aging Med Healthc.

[33] Messina M. (2016). Soy and health update: evaluation of the clinical and epidemiologic literature. Nutrients.

[34] Messina M., Duncan A., Messina V., Lynch H., Kiel J., Erdman J.W. (2022). The health effects of soy: a reference guide for health professionals. Front Nutr.

[35] Suthuvoravut U., Takahashi K., Murayama H., Tanaka T., Akishita M., Iijima K. (2020). Association between traditional Japanese diet washoku and sarcopenia in community-dwelling older adults: findings from the Kashiwa study. J Nutr Health Aging.

[36] Talegawkar S.A., Bandinelli S., Bandeen-Roche K., Chen P., Milaneschi Y., Tanaka T. (2012). A higher adherence to a Mediterranean-style diet is inversely associated with the development of frailty in community-dwelling elderly men and women. J Nutr.

[37] León-Muñoz L.M., Guallar-Castillón P., López-García E., Rodríguez-Artalejo F. (2014). Mediterranean diet and risk of frailty in community-dwelling older adults. J Am Med Dir Assoc.

[38] Kojima G., Taniguchi Y., Urano T. (2022). Fruit and vegetable consumption and incident frailty in older adults: a systematic review and meta-analysis. J Frailty Aging.

[39] Kojima G., Iliffe S., Jivraj S., Walters K. (2020). Fruit and vegetable consumption and incident prefrailty and frailty in community-dwelling older people: the English longitudinal study of ageing. Nutrients.

[40] Lyu W., Tanaka T., Bo-Kyung S., Yoshizawa Y., Akishita M., Iijima K. (2024). Integrated effects of nutrition-related, physical, and social factors on frailty among community-dwelling older adults: a 7-year follow-up from the Kashiwa cohort study. Geriatr Gerontol Int.

[41] Weening-Dijksterhuis E., de Greef M.H., Scherder E.J., Slaets J.P., van der Schans C.P. (2011). Frail institutionalized older persons: a comprehensive review on physical exercise, physical fitness, activities of daily living, and quality-of-life. Am J Phys Med Rehabil.

[42] Hirose T., Sawaya Y., Ishizaka M., Hashimoto N., Kubo A., Urano T. (2025). Frailty incidence proportion is more influenced by age than by time period: a retrospective study from 2012 to 2022. Geriatr Gerontol Int.

[43] Dent E., Morley J.E., Cruz-Jentoft A.J., Woodhouse L., Rodríguez-Mañas L., Fried L.P. (2019). Physical frailty: ICFSR international clinical practice guidelines for identification and management. J Nutr Health Aging.

[44] Varadhan R., Chaves P.H., Lipsitz L.A., Stein P.K., Tian J., Windham B.G. (2009). Frailty and impaired cardiac autonomic control: new insights from principal components aggregation of traditional heart rate variability indices. J Gerontol A Biol Sci Med Sci.

