# Supplementary material for: Association between frailty recovery and dietary variety among community-dwelling older Japanese adults: a longitudinal study from 2023 to 2024
Source: J Nutr Health Aging. 2026 Jan 21;30(3):100783. doi: 10.1016/j.jnha.2026.100783 (PMC12859231; doi:10.1016/j.jnha.2026.100783)
Supplement: Supplementary file 1 [file mmc1.docx]

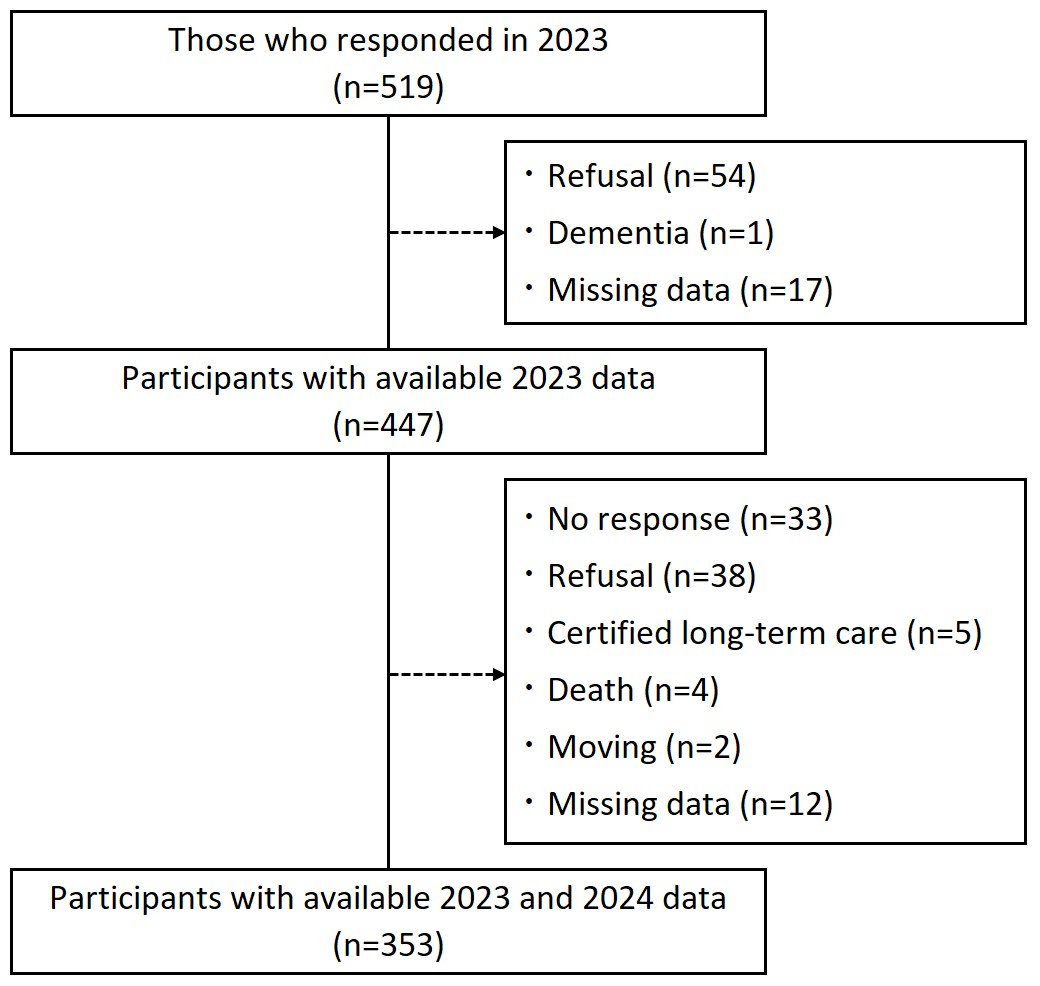


**Figure S1**. Flowchart of participant intake

**Table S1.** Comparison of the baseline characteristics between the robust-maintained/-recovered and others groups

|  | Robust-maintained/-recovered group (n=184) | Others (n=169) | P value |
| --- | --- | --- | --- |
| Age |  |  |  |
| 73 years old | 129 (70.1) | 99 (58.6) | 0.024* |
| 78 years old | 55 (29.9) | 70 (41.4) |  |
| Sex |  |  |  |
| Male | 96 (52.2) | 87 (51.5) | 0.896 |
| Female | 88 (47.8) | 82 (48.5) |  |
| Height (cm) | 159.2 ± 8.7 | 158.2 ± 8.2 | 0.264 |
| Weight (kg) | 59.7 ± 10.8 | 58.0 ± 9.7 | 0.125 |
| Body mass index (kg/m^2^) | 23.5 ± 3.3 | 23.1 ± 3.0 | 0.305 |
| Living alone | 19 (10.3) | 20 (11.8) | 0.652 |
| Comorbidity |  |  |  |
| Hypertension | 79 (42.9) | 88 (52.1) | 0.086 |
| Hyperlipidemia | 34 (18.5) | 32 (18.9) | 0.912 |
| Cerebrovascular disease | 0 (0) | 0 (0) | – |
| Cancer | 4 (2.2) | 10 (5.9) | 0.072 |
| Hobbies | 110 (59.8) | 72 (42.6) | 0.001* |
| Community activities | 89 (48.4) | 75 (44.4) | 0.453 |

n (%). *: p < 0.05．

Robust-maintained/-recovered group: Individuals who were classified as robust in both 2023 and 2024, as well as those who were classified as pre-frailty or frailty in 2023 and transitioned to robust in 2024.

Others: Individuals other than those in the robust-maintained/-recovered group.

The proportions of individuals living alone, comorbidities, hobbies, and participation in community activities have also been reported.

**Table S2**. Association between the robust-maintained/-recovered group and longitudinal nutritional status based on the Dietary Variety Score

|  | n | Crude model | | | Model Ⅰ | | |
| --- | --- | --- | --- | --- | --- | --- | --- |
|  |  | OR | 95%CI (Min–Max) | P value | OR | 95%CI (Min–Max) | P value |
| Low DVS–Low DVS | 151 | Reference | | | Reference | | |
| Low DVS–High DVS | 39 | 0.92 | 0.45–1.88 | 0.820 | 0.89 | 0.43–1.86 | 0.759 |
| High DVS–Low DVS | 41 | 1.14 | 0.57–2.29 | 0.706 | 1.28 | 0.62–2.62 | 0.502 |
| High DVS–High DVS | 122 | 2.92 | 1.77–4.82 | <0.001* | 2.86 | 1.69–4.84 | <0.001* |

CI, confidence interval; DVS: Dietary Variety Score, Max, maximum; Min, minimum; OR, odds ratio. *: p<0.05．

The dependent variables were defined as 0 (others) or 1 (robust-maintained/-recovered).

The independent variables were the baseline DVS–follow-up DVS.

High DVS: DVS ≥ 4; low DVS: DVS ≤ 3.

Low DVS–Low DVS: Low DVS in both 2023 and 2024.

Low DVS–High DVS: Low DVS in 2023 and high DVS in 2024.

High DVS–Low DVS: High DVS in 2023 but low DVS in 2024.

High DVS–High DVS: High DVS in both 2023 and 2024.

Model I (adjusted variables): Age, sex, body mass index, living alone, hypertension, hyperlipidemia, cancer, hobbies, and community activities.

Robust-maintained/-recovered group: Individuals who were classified as robust in both 2023 and 2024, as well as those who were classified as pre-frailty or frailty in 2023 and transitioned to robust in 2024.

Others: Individuals other than those in the robust-maintained/-recovered group.

**Table S3**. Comparison of baseline characteristics between frailty-persistent/new-onset and other groups

|  | Frailty-persistent/new-onset group (n=64) | Others (n=289) | P value |
| --- | --- | --- | --- |
| Age |  |  |  |
| 73 years old | 38 (59.4) | 190 (65.7) | 0.335 |
| 78 years old | 26 (40.6) | 99 (34.3) |  |
| Sex |  |  |  |
| Male | 39 (60.9) | 144 (49.8) | 0.107 |
| Female | 25 (39.1) | 145 (50.2) |  |
| Height (cm) | 160.3 ± 8.5 | 158.3 ± 8.5 | 0.103 |
| Weight (kg) | 59.3 ± 8.8 | 58.8 ± 10.6 | 0.658 |
| Body mass index (kg/m^2^) | 23.1 ± 2.6 | 23.3 ± 3.3 | 0.443 |
| Living alone | 5 (7.8) | 34 (11.8) | 0.361 |
| Comorbidity |  |  |  |
| Hypertension | 31 (48.4) | 136 (47.1) | 0.842 |
| Hyperlipidemia | 10 (15.6) | 56 (19.4) | 0.486 |
| Cerebrovascular disease | 0 (0) | 0 (0) | – |
| Cancer | 1 (1.6) | 13 (4.5) | 0.480 |
| Hobbies | 20 (31.3) | 162 (56.1) | <0.001* |
| Community Activities | 20 (31.3) | 144 (49.8) | 0.007* |

n (%). *: p<0.05．

Frailty-persistent/new-onset group: Those who met the criteria for frailty in both 2023 and 2024, and those who were classified as robust or pre-frailty in 2023 but deteriorated to frailty in 2024.

Others: Individuals other than those in the frailty persistent/new-onset group.

The proportions of individuals living alone, comorbidities, hobbies, and participation in community activities were also reported.

**Table S4**. Comparison of the intake proportions of the 10 food groups and total Dietary Variety Scores between the groups

|  | | Frailty-persistent/new-onset group (n=64) | Others (n=289) | P value | |
| --- | --- | --- | --- | --- | --- |
| 1 | Fish/shellfish |  |  |  | |
|  | Almost every day/Once every 2 days/1–2 days a week/Hardly ever | 17.2/28.1/50.0/4.7 | 28.7/33.9/36.0/1.4 | 0.029* | |
| 2 | Meat |  |  |  | |
|  | Almost every day/Once every 2 days/1–2 days a week/Hardly ever | 17.2/43.8/37.5/1.6 | 29.8/42.2/26.6/1.4 | 0.119 | |
| 3 | Eggs |  |  |  | |
|  | Almost every day/Once every 2 days/1–2 days a week/Hardly ever | 25.0/34.4/35.9/4.7 | 38.1/34.3/26.0/1.7 | 0.072 | |
| 4 | Milk |  |  |  | |
|  | Almost every day/Once every 2 days/1–2 days a week/Hardly ever | 37.5/10.9/23.4/28.1 | 47.1/11.8/19.4/21.8 | 0.488 | |
| 5 | Soybean products |  |  |  | |
|  | Almost every day/Once every 2 days/1–2 days a week/Hardly ever | 40.6/28.1/28.1/3.1 | 60.2/21.8/17.6/0.3 | 0.007* | |
| 6 | Green and yellow vegetables |  |  |  | |
|  | Almost every day/Once every 2 days/1–2 days a week/Hardly ever | 54.7/26.6/17.2/1.6 | 69.9/17.3/11.8/1.0 | 0.094 | |
| 7 | Seaweeds |  |  |  | |
|  | Almost every day/Once every 2 days/1–2 days a week/Hardly ever | 12.5/34.4/40.6/12.5 | 22.5/29.1/43.9/4.5 | 0.039* | |
| 8 | Potatoes |  |  |  | |
|  | Almost every day/Once every 2 days/1–2 days a week/Hardly ever | 7.8/14.1/59.4/18.8 | 12.5/26.0/56.1/5.5 | 0.001* | |
| 9 | Fruits |  |  |  | |
|  | Almost every day/Once every 2 days/1–2 days a week/Hardly ever | 21.9/21.9/34.4/21.9 | 33.2/20.1/37.7/9.0 | 0.018* | |
| 10 | Fats/oils |  |  |  | |
|  | Almost every day/Once every 2 days/1–2 days a week/Hardly ever | 32.8/37.5/29.7/0.0 | 33.2/30.4/33.9/2.4 | 0.540 | |
| DVS (total points) | | 3.0 [1.0–4.0] | 3.0 [2.0–5.0] | | 0.002* |

DVS: Dietary Variety Score. %. Median [25th–75th percentile]. *: p<0.05.

Frailty-persistent/new-onset group: Those who met the criteria for frailty in both 2023 and 2024, and those who were classified as robust or pre-frailty in 2023 but deteriorated to frailty in 2024.

Others: Individuals other than those in the frailty persistent/new-onset group.

**Table S5**. Association between the frailty-persistent/new-onset group and the baseline Dietary Variety Score

|  | n | Crude model | | | Model Ⅰ | | |
| --- | --- | --- | --- | --- | --- | --- | --- |
|  |  | OR | 95%CI (Min–Max) | P value | OR | 95%CI (Min–Max) | P value |
| High DVS (Baseline) | 163 | Ref | | | Ref | | |
| Low DVS (Baseline) | 190 | 1.98 | 1.12–3.50 | 0.019* | 1.78 | 0.98–3.24 | 0.057 |

CI: confidence interval, DVS: Dietary Variety Score, Max: maximum, Min: minimum, OR: odds ratio. *: p<0.05.

The dependent variables were defined as 0 (others) or 1 (frailty-persistent/new-onset).

The independent variables were defined as 0 (High DVS) or 1 (Low DVS).

High DVS: DVS ≥ 4; low DVS: DVS ≤ 3.

Model I (adjusted variables): Age, sex, body mass index, living alone, hypertension, hyperlipidemia, cancer, hobbies, and community activities.

Frailty-persistent/new-onset group: Those who met the criteria for frailty in both 2023 and 2024, and those who were classified as robust or pre-frailty in 2023 but deteriorated to frailty in 2024.

Others: Individuals other than those in the frailty persistent/new-onset group.

**Table S6**. Association between the frailty-persistent/new-onset group and longitudinal nutritional status based on the Dietary Variety Score

|  | n | Crude model | | | Model Ⅰ | | |
| --- | --- | --- | --- | --- | --- | --- | --- |
|  |  | OR | 95%CI (Min–Max) | P value | OR | 95%CI (Min–Max) | P value |
| High DVS–High DVS | 122 | Reference | | | Reference | | |
| High DVS–Low DVS | 41 | 2.58 | 1.00–6.66 | 0.051 | 2.24 | 0.82–6.11 | 0.116 |
| Low DVS–High DVS | 39 | 2.01 | 0.73–5.52 | 0.178 | 1.78 | 0.62–5.16 | 0.285 |
| Low DVS–Low DVS | 151 | 2.87 | 1.42–5.80 | 0.003* | 2.51 | 1.20–5.24 | 0.014* |

CI, confidence interval; Max, maximum; Min, minimum; OR, odds ratio. *: p < 0.05.

Dependent variables were defined as 0 (others) or 1 (frailty persistent/new onset).

The independent variables were the baseline DVS–follow-up DVS.

High DVS: DVS ≥ 4; low DVS: DVS ≤ 3.

Low DVS–Low DVS: Low DVS in both 2023 and 2024.

Low DVS–High DVS: Low DVS in 2023 and high DVS in 2024.

High DVS–Low DVS: High DVS in 2023 but low DVS in 2024.

High DVS–High DVS: High DVS in both 2023 and 2024.

Model I (adjusted variables): Age, sex, body mass index, living alone, hypertension, hyperlipidemia, cancer, hobbies, and community activities.

Frailty-persistent/new-onset group: Those who met the criteria for frailty in both 2023 and 2024, and those who were classified as robust or pre-frailty in 2023 but deteriorated to frailty in 2024.

Others: Individuals other than the Frailty persistent/new-onset group.

**Table S7.** Association between the frailty-persistent/new-onset group and the ten individual food items of the Dietary Variety Score at baseline

|  | Crude model | | | Model Ⅰ | | |
| --- | --- | --- | --- | --- | --- | --- |
|  | OR | 95%CI (Min–Max) | P value | OR | 95%CI (Min–Max) | P value |
| Soybean products (Baseline) | 0.45 | 0.26–0.79 | 0.005* | 0.53 | 0.30–0.95 | 0.033* |

CI: confidence interval, Max: maximum, Min: minimum, OR: odds ratio. *: p<0.05.

The dependent variables were defined as 0 (others) or 1 (frailty-persistent/new-onset).

The independent variables were the ten food items of the Dietary Variety Score, which were entered into the model using a stepwise method. Each food item was coded as 0 (once every 2 days, 1–2 days per week, or hardly ever) or 1 (almost every day).

Model I (adjusted variables): Age, sex, body mass index, living alone, hypertension, hyperlipidemia, cancer, hobbies, and community activities.

Frailty-persistent/new-onset group: Those who met the criteria for frailty in both 2023 and 2024, and those who were classified as robust or pre-frailty in 2023 but deteriorated to frailty in 2024.

Others: Individuals other than those in the frailty-persistent/new-onset group.
